# On the Action of Digitalis, and Its Uses in Diseases of the Heart

**Published:** 1845-01

**Authors:** 


### On the Action of Digitalis and its Uses in Diseases of the Heart. By *William Munk*, M.D. (Guy's Hospital Reports, October, 1844.)

This practical paper is founded upon upwards of 400 observations, which were made during five years of dispensary practice. The tincture has been found the most successful form of preparation as regards the effect produced upon the action of the heart, while the infusion is incomparably superior as a diuretic; and, from want of attention to this distinction, discrepant opinions as to the utility of the medicine have doubtless arisen. The powder, used alone, Dr. Munk considers as worthless, and although in combination (with mercury and squill) it forms a valuable diuretic, it cannot be even so employed as a sedative.

The action of digitalis upon the heart is manifested in two ways; by the exertion of a depressing influence, and as an antispasmodic. Hypertrophy of the organ, whether simple or complicated with other disease, causing increased impulse, may be benefited by the *depressing* influence of digitalis, which is best obtained by giving the uncombined tincture, in tolerably full doses, at intervals of eight, ten, or twelve hours. The *antispasmodic* influence, acting so beneficially in the irritable condition of the heart manifested by palpitations, irregularity, &c., is that which is usually sought from digitalis. Dr. Munk does not agree with those writers who state the action of digitalis upon the heart to be uncertain. Its operation is as certain, in properly-selected cases, as that of other medicines, and may be maintained with safety.

There are, however, circumstances under which this medicine cannot be exhibited usefully or safely. Thus, in a plethoric state of the system, its employment must be deferred until blood-letting or other evacuants, for which it is no substitute, have played their parts. An inflammatory, or sub-inflammatory, condition of the gastric and intestinal mucous membrane, seems to prevent the action of digitalis upon the heart; and increased irritation results if it be persisted in. Such complication of lesion of the heart and gastric derangement is by no means rare, and in such cases prussic acid is the appropriate medicine. Quietude of mind and body much favour the action of digitalis. The recumbent posture is very adjuvatory to its depressent action; and Dr. Lombard truly observes that it is rarely efficacious in those who take much exercise, or whose attention is much occupied during its use. Dr. Munk gives ♏ x. ad xxx. every eight, ten, or twelve hours, and is rarely disappointed. He does not reduce the pulse, which is to be carefully watched, below 60 in the adult, and thus derives the beneficial without risking the production of the dangerous effects.

Digitalis rarely acts as a *diuretic* when its influence upon the heart is marked, and *vice versâ*. The author quite concurs in the high opinion Withering entertained of its power of increasing the flow of urine, which is seldom accomplished by any other drug after its failure. It is not to the robust, florid, or wiry-pulsed, but to the enfeebled, shattered, condition of the system that digitalis is applicable. "If the pulse be feeble or intermitting, the countenance pale, the lips livid, the skin cold, the swollen belly soft and fluctuating, or the anasarcous limbs readily pitting on pressure, we may expect the diuretic effects to follow in a kindly manner. These remarks were penned, it is true, in reference to dropsy, from whatever cause arising; but, *mutatis mutandis*, they are equally applicable to all cases in which the diuretic operation of foxglove is required." In diseases of the heart, a diuresis is frequently a valuable means of preventing effusions by diminishing congestion, or of producing their absorption if they have already occurred; but whether digitalis be the appropriate remedy or not, depends in chief upon whether a sthenic or asthenic condition of the system prevail. Thus, in the case of hypertrophy, it is seldom appropriate, while in dilatation it is usually the best of diuretics. Valvular disease is that in which digitalis proves most useful, except in cases in which this is complicated with hypertrophy. The *infusion* is to be given in doses of ℥ss. to ℥j. every six or eight hours. With a view of preventing the sedative operation of the drug, moderate exercise, short of diaphoresis, should when possible be taken. A moderate quantity of drink may be given; and the loins must be covered with a double roll of flannel, or, as recommended by Lombard, a stimulating plaister may be applied to them.

Dr. Munk believes that untoward and fatal effects, resulting from the continued employment of this medicine, are "exceedingly rare;" and cites the opinions of Drs. Holland and Pereira as confirmatory of his own.

"It has only occurred to me to see the slighter and less portentous of these symptoms as a consequence of fox-glove; such as, inequality or intermittence of the pulse, loss of appetite, and frontal headache: either or all of which have at once subsided on discontinuing the medicine. I believe that such symptoms will only occur when the drug fails to act in a normal manner as a sedative or a diuretic. If either of these effects are once obtained in a kindly manner, I then consider my patient safe from the poisonous operation of the drug. If, on the contrary, it does not evidence its usual effects within a few days, the medicine, I believe, accumulates in the system, and the patient is in danger of experiencing its poisonous influence. I am therefore in the habit of prescribing it for a week: and, if within that period, I perceive neither sedative or diuretic effects, I then invariably desist from its administration. Let these effects, however, be once kindly induced, and the medicine may then be continued with safety for a considerable period. In no one instance have I seen a bad effect follow the use of digitalis where the first consequences of its exhibition were the removal or material alleviation of prominent or distressing cardiac symptoms, whether this has been brought about by its operation as a sedative or as a diuretic." 310.

